# Colonization and transmission of *Staphylococcus aureus* in schools: a citizen science project

**DOI:** 10.1099/mgen.0.000993

**Published:** 2023-04-19

**Authors:** Andries J. van Tonder, Frances McCullagh, Hanan McKeand, Sue Thaw, Katie Bellis, Claire Raisen, Liz Lay, Dinesh Aggarwal, Mark Holmes, Julian Parkhill, Ewan M. Harrison, Adam Kucharski, Andrew Conlan

**Affiliations:** ^1^​ Department of Veterinary Medicine, University of Cambridge, Cambridge, UK; ^2^​ Cottenham Village College, Cottenham, UK; ^3^​ St Bede's Inter-Church School, Cambridge, UK; ^4^​ Wellcome Sanger Institute, Hinxton, UK; ^5^​ Department of Medicine, University of Cambridge, Cambridge, UK; ^6^​ Department of Public Health and Primary Care, University of Cambridge, Cambridge, UK; ^7^​ Centre for Mathematical Modelling of Infectious Diseases, London School of Hygiene and Tropical Medicine, London, UK

**Keywords:** MRSA, schools, social network analysis, transmission, WGS

## Abstract

Aggregation of children in schools has been established to be a key driver of transmission of infectious diseases. Mathematical models of transmission used to predict the impact of control measures, such as vaccination and testing, commonly depend on self-reported contact data. However, the link between self-reported social contacts and pathogen transmission has not been well described. To address this, we used *Staphylococcus aureus* as a model organism to track transmission within two secondary schools in England and test for associations between self-reported social contacts, test positivity and the bacterial strain collected from the same students. Students filled out a social contact survey and their *S. aureus* colonization status was ascertained through self-administered swabs from which isolates were sequenced. Isolates from the local community were also sequenced to assess the representativeness of school isolates. A low frequency of genome-linked transmission precluded a formal analysis of links between genomic and social networks, suggesting that *S. aureus* transmission within schools is too rare to make it a viable tool for this purpose. Whilst we found no evidence that schools are an important route of transmission, increased colonization rates found within schools imply that school-age children may be an important source of community transmission.

## Data Summary

1. Supplementary Files 1 and 2 (available with the online version of this article).
2. Raw sequencing data for all sequenced isolates have been deposited in the European Nucleotide Archive (ENA) under project PRJEB28206. Accession numbers are detailed in Supplementary File 1.
3. The R code used to analyse the data in this paper is available on GitHub: https://github.com/MonkeyMyshkin/SchoolsAMR.

**Impact Statement**

School-aged children have been shown to be an important driver in the transmission of infectious disease. Disease dynamic models that are used to inform policy decisions such as vaccine schedules and contact tracing routinely depend on self-reported social contact data which measure the relative weight of contact between different groups as a proxy measure of transmission risk. However, validation of the link between self-reported social contacts and the transmission of directly transmitted pathogens is still lacking and, to our knowledge, no study has previously attempted to use microbial genomics to track transmission networks within a school environment and compare these with social networks. We used *Staphylococcus aureus* as a model organism to track transmission of this commensal and opportunistic pathogen within the school environment, an age group that has rarely been studied with respect to *S. aureus* prevalence and transmission. Whilst a low frequency of genome-linked transmission within the school precluded a formal analysis of links between genomic and social networks, it has important implications for our understanding of the ecology and community transmission of *S. aureus *. The diversity of strains found within schools was indistinguishable from an independent sample set from the general community, but with an overall higher carriage rate in the school-age children, suggesting that children may well be an important core group in transmission, but the setting for that transmission is within the community rather than the school environment. The study also provided important insights into the feasibility of carrying out such studies in schools beyond the suitability of *S. aureus* as a model organism. Despite intensive efforts in terms of engagement activities with schools, we achieved far lower rates of participation than in previous studies where biological samples (swabs) were not taken, though participation rates were still higher than in subsequent studies in schools for SARS-CoV-2 (severe acute respiratory syndrome coronavirus 2). However, the difference between our latest study and those for SARS-CoV-2 is far smaller than for earlier studies that only collected social contact data. This suggests that reticence to perform swabbing and provide biological samples limits participation rates in these kinds of studies in schools, which must be factored into future study designs.

## Introduction

School-aged children have been shown to be an important core group in the transmission of infectious disease, most famously with respect to classical childhood infections [1, 2], but also for more general community-acquired infections such as influenza and SARS-CoV-2 (severe acute respiratory syndrome coronavirus 2) [3, 4]. In the case of influenza, the introduction of vaccination for school-age children in the UK was partially motivated by the expected protective benefit for older vulnerable risk groups resulting from the reduction in levels of transmission within the community from disease dynamic transmission models [5]. Disease dynamic models that are used to inform policy decisions on vaccine schedules, contact tracing and non-pharmaceutical interventions now routinely depend on self-reported social contact data which measure the relative weight of contact between different groups – particularly with respect to age [6–8] – as a proxy measure of transmission risk. However, due to acute infections being difficult to sample accurately and immunological markers not being a direct measure of time of infection, empirical validation of the link between self-reported social contacts and the transmission of directly transmitted pathogens is still lacking. In this pilot study, we used *Staphylococcus aureus* as a model organism to attempt to validate this link by using microbial genomics to track the transmission of *S. aureus* [9, 10] within a school environment and test for associations between the self-reported social contact network and the genetic similarity of recovered bacteria.

*S. aureus* is a Gram-positive commensal bacterium and opportunistic pathogen, and causes a broad range of infections in humans, from minor skin and soft tissue infections to life-threatening bloodstream infections [11]. The treatment of *S. aureus* infections is compounded by resistance to β-lactams in meticillin-resistant *S. aureus* (MRSA), and the development of multidrug-resistant strains [12]. Though *S. aureus* is capable of colonizing multiple body sites, the nose is regarded as the primary reservoir, with ~30 % of people being nasal carriers [13, 14]. Nasal carriage is of clinical importance as it is a risk factor for staphylococcal infections [15], and as such the detection of MRSA carriers and their subsequent decolonization is a key component of hospital infection control. Serious clinical infections in humans are rare relative to the prevalence of carriage, but the severity of outcomes means they are of significant clinical importance [11]. Data on the prevalence of *S. aureus* within school-age children in the UK have been exceptionally limited [13, 14]; thus, a secondary objective of our study was to quantify any differences in prevalence of carriage and diversity of lineages within our schools compared with the sympatric adult population.

Phylodynamic models [16], which unify the evolutionary and epidemiological dynamics of infectious agents, provide the promise of inferring the transmission network within a population [17]. However, inference from genomic data alone can be extremely sensitive to under-, or un-representative, sampling of the host population [18]. As a consequence, there has been considerable interest in the use of social contact networks to support or strengthen inference from sequence data [19]. Outside of infections with highly specific routes of transmission, such as sexually transmitted diseases, comparatively less attention has been paid to identifying the types of social interaction most closely correlated to transmission of communicable infections [19] or specifically comparing the contact patterns within schools with transmission [20]. To our knowledge, no study has previously attempted to use microbial genomics to track transmission networks within a school environment and compare with social networks, though in the recent COVID-19 pandemic this has been done in universities [21].

To this end, we carried out a pilot study on the carriage of *S. aureus* within two secondary schools (specifically year groups 7–10 corresponding to pupils aged 10–15 years old) in England between March 2018 and July 2019. Colonization status of participating pupils was ascertained through self-administered nasal swabs [22] from which isolates were cultured and sequenced. Taking a public engagement approach that has previously delivered exceptionally high participation rates of >90 % [23–26], we recruited classes within our study schools as citizen scientists to inform the design and delivery of our study within their own school.

## Methods

### Schools

After an open call for interest, two schools from Cambridgeshire were chosen as partners for the project based on their geographical separation and convenience for visits from Cambridge. The project was then run over the course of an entire school year, with the research team working together with student scientists in each school to plan, deliver and carry out preliminary analyses of the collected data. The research team delivered four lessons to each school on epidemic models, scientific ethics, bacterial genome sequencing and social network analysis. To attempt to further boost engagement outside of the student scientists’ own classes, the principal investigator also delivered short presentations during morning assemblies of the whole school on the topic of infectious disease epidemiology.

The study was carried out under approval from the Department of Veterinary Medicine Ethics Committee (CR253) and the University of Cambridge Human Biology Research Ethics Committee (HBREC.2018.29), and repeated over the course of two school years (2017/2018 and 2018/2019). To protect the identity of participants in the study, we will refer to these schools as School 1 and School 2. Both are state-funded schools of a similar size, with School 1 located in a village outside of the city and School 2 within Cambridge itself.

### Study population

The trial protocol was varied slightly both between schools and years, in line with the practical constraints within each school and in the second year of the project (Year 2) to attempt to improve unexpectedly low recruitment rates. Students from school years 9 to 11 were initially eligible for inclusion within the study; however, for logistical reasons School 2 could only target one year group (year 10 in the first year, year 9 in the second), whereas School 1 was able to target a broader range of school years (years 7–10 in the first year and years 7–9 in the second). In both years of the study, parents of eligible students were sent letters and consent forms (physical letters in Year 1, e-mails with links to an electronic consent from in Year 2). Pupils were also asked to give verbal assent to take part in the study when data and samples were collected. Administration of contact surveys and the collection of samples was organized and carried out by the citizen science teams within their own school before being delivered to the research team for processing.

### Exclusion criteria and self-administered swabbing

Pupils with parental consent were further screened and excluded from the study if they met the following criteria: nasal abnormalities or medical conditions including nosebleeds, nasal polyps or rhinitis, whether they had undergone nasal surgery in the year prior to the study or else suffered a nasal fracture in the year prior to the study. Following training, the students then self-administered nasal swabs at two different timepoints (60–90 days apart); students who refused nasal swabs were excluded.

The nasal swabs were placed in a tube containing 5 ml Mueller–Hinton broth supplemented with 6.5 % NaCl and incubated overnight at 37 °C. An aliquot of 50 µl of the enrichment broth was spread on chromogenic Staph Brilliance 24 agar plates and Brilliance MRSA 2 agar plates (Oxoid) and incubated overnight at 37 °C. Blue colonies – putative *S. aureus* colonies – were sub-cultured onto Staph Brilliance agar and *S. aureus* confirmed by multiplex PCR to detect *mecA*, *mecC* and *femB* (* S. aureus *-specific target) genes [27]. Genomic DNA was extracted from overnight cultures grown in tryptic soy broth (TSB) using the Epicenter MasterPure Gram positive DNA purification kit, according to the manufacturer's protocol, except that a pre-treatment with 10mg/ml lysozyme to more efficiently lyse the cells was applied before extraction. The extracted DNA was sequenced at the Wellcome Sanger Institute using the Illumina HiSeq X10 platform to generate 2×150 bp paired-end reads.

### Social contact survey

Alongside the nasal swabbing, students participating in the study were asked to fill out a social contact survey (Supplementary File 2); students who didn’t complete the survey were also excluded. Taking the same structure used in previous studies [23, 26], this questionnaire consists of a set of core questions asking students to report the names of students they spend the most time with from their own year group (up to six names) and outside of their year group (up to four names), along with their class and a small number of additional questions chosen by the student scientists. For this project, the pupils from both schools chose to ask whether students shared drinks with their friends.

In Year 1, following the successful methodology used in previous projects, social contact data were collected by a paper questionnaire. To protect the students’ identities, these forms were digitized and anonymized within the grounds of each school by the research team to allow checking of names of contacts with the school register. In Year 2, contact data – and parental consent – were collected through an online web-form on the Qualtrics platform (https://www.qualtrics.com/security-statement/). This change avoided the necessity to digitize paper forms within the schools, with the disadvantage of losing access to the school registers. A unique list of participants and their contacts was manually curated, guided by an initial matching of names based on fuzzy matching (lowest common substring method) using the ‘stringdist’ [28] package in R [29]. This list of unique participants within each school was then linked to the swab IDs (where these were provided), with dummy IDs generated for all reported contacts that were not part of the carriage study.

While the change in protocol had benefits for data curation, the primary motivation of moving to electronic consent and data capture was to make participation (and granting of consent in particular) easier, given that both schools primarily communicated with students through e-mail and online portals. Reporting of student’s classes was inconsistently coded by students from both schools using both methods, so we chose not to analyse that here.

### Descriptive analysis of *S. aureus* colonization data

Descriptive analyses of the public anonymized data set linking social contacts and carriage status was carried out using the R statistical language. Prevalence within sub-groups was calculated using the ‘prevalence’ package [30] with exact (Clopper–Pearson) confidence intervals (CIs). The association of carriage with school, year of study, year group, gender of participant and whether they share drinks with their friends (*shares drinks*) was explored using a logistic regression model with the response variable for individual students defined as positive if *S. aureus* was isolated from either of their two swabs. Risk factors were selected by purposeful selection [31] with a generous initial univariate screen (90 % significance level), followed by forwards and backwards selection by Akaike information criterion (AIC).

### Social network analysis

Social networks within the school were visualized and analysed using igraph [32], tidygraph [33] and ggraph [34]. As in our previously published work [26], we define an edge between two pupils when at least one names the other, rather than requiring that both pupils name each other (mutual link) as we used elsewhere for British primary schools [23]. This definition is less likely to miss social links (more sensitive) but is also more liable to misreporting through either aspirational naming of contacts [35] or simply errors in recording (less specific).

To compare the structure of the observed social networks between rounds of the study and schools, we calculated a set of summary statistics: the *clustering coefficient* (transitivity) defined as the ratio of triangles within the network divided by the number of connected triples; *reciprocity* defined as the fraction of mutual links where both students named each other; *mean distance* defined as the mean path length within the network (shortest path between each node); the number and mean size of communities as defined by the ‘walktrap’ algorithm [36]. Finally, to explore nodal variables relationship with the network structure, we calculated the (nominal) assortativity with respect to year, class, gender, colonization status and whether students share drinks with their friends [37]. For each network statistic, CIs were calculated by constructing 10 000 bootstrap networks by randomly sampling edges (with replacement) from the list of edges recorded for each school/round.

### Genomic analyses

Quality control metrics were generated for the raw sequence data using fastQC [38] and species classification for each read was performed using Kraken and Bracken [39, 40]. Samples with <70 % reads matching to *S. aureus* were removed from the analyses. Annotated assemblies were produced using a previously described pipeline [41]. Briefly, for each sample, multiple assemblies were created using VelvetOptimiser v2.2.5 and Velvet v1.2 [42]. The assembly with the best N50 value (The sum of the lengths of all contigs of size N50 or longer contain at least 50 percent of the total genome sequence) was chosen and the contigs were then scaffolded using sspace [43] and sequence gaps closed using GapFiller [44]. Annotation was performed using prokka v1.5 [45] and a genus-specific database from RefSeq. Sequence types (STs) and antibiotic-resistance profiles (from the CARD database) were assigned to the assemblies using ariba [46]. Core-genome alignments were generated for each subset of data using Panaroo and a core-genome threshold of 98 % [47]. Variant sites were extracted from the core-genome alignments using snp-sites [48] and phylogenetic trees were reconstructed using iq-tree, incorporating invariant sites from the alignment, running 1000 ultrafast bootstraps and using the built-in model testing to determine the best phylogenetic model [49]. Pairwise SNP distances between each pair of isolates were calculated using pairsnp [50]. A threshold for determining putative transmission was determined by plotting the distribution of pairwise SNP distances between isolates from the same patient, i.e. within-host diversity (Fig. S1). The majority of within-host isolates were within 50 SNPs of each other, so this threshold was used to calculate putative transmission clusters using igraph [32].

## Results

### Recruitment rates

Despite enthusiastic support from school management and the classes working directly with the research team, recruitment rates in the first year were low at 23 and 31 % for School 1 and 2, respectively (Table 1). Only one parent over the 2 years contacted the principal investigator directly asking for a child specifically not to be included in the project, but did not provide their reason. While a small number of parents contacted a teacher with concerns about confidentiality, the vast majority simply did not respond making systematic assessment of their concerns challenging.

**Table 1.** Summary of sample size and prevalence of *S. aureus* at both schools by year

School	Year	Round	Date	Positive	Samples	Target population	% Recruitment rate	% Overall prevalence (95 % CI)	% Male prevalence (95 % CI)	% Female prevalence (95 % CI)
1	1	1	Mar 18	73	183	793	23	40 (33–47)	48 (37–59)	33 (24–43)
		2	Apr 18	71	183	793		39 (32–46)	53 (42–64)	27 (18–37)
	2	1	May 19	102	185	500	37	55 (48–62)	64 (53–73)	46 (36–57)
		2	Jul 19	89	185	500		48 (41–56)	55 (45–66)	41 (30–51)
2	1	1	Mar 18	12	66	160	31	18 (10–30)	28 (12–49)	9 (1–28)
		2	Apr 18	11	66	160		17 (9–28)	28 (12–49)	13 (3–34)
	2	1	May 19	8	20	190	17	40 (19–64)	50 (21–79)	25 (3–65)
		2	Jul 19	6	20	190		30 (12–54)	33 (10–65)	25 (3–65)

Through exit interviews after Year 1 with the teachers and pupils, there was anecdotal evidence that some children were reluctant to take part due to concerns about the physical process of taking a swab and potential embarrassment of doing this around their peers. To reduce this concern, School 1 employed screens in the second year for children to use when collecting the sample. Both schools also recommended moving to an online consent process rather than physical letters and forms, as the majority of school communication with parents was done using a mixture of e-mail and online portals. As a further motivation for involvement, a prize draw for participating students was also introduced in the second year where two students from each school could win a £25 gift voucher. Despite these additional efforts, recruitment only increased by 14 % in the second round of data collection for School 1 and actually fell for School 2 despite the same measures being taken (Table 1).

### Prevalence and risk factors for carriage of *S. aureus*

The 2 years of survey data reveal a dynamically changing rate of carriage within each school, with consistent differences between schools and gender groups between the 2 years. In common with surveys of adults, carriage rates are higher in boys compared to girls (difference of 14–25 %; Table 1). The total rates of carriage increased for both schools in Year 2, from 39 to 57 % at School 1 (Table 1) and from 22 to 36 % at School 2. The carriage rates in School 1 from both years are significantly higher than the estimated point prevalence of 36 % (35–36 %, 95 % CI) found in the independent unpublished CARRIAGE study of the local adult population in England (E. M. Harrison, personal communication).

To explore the association between carriage and known risk factors measured by the questionnaire study, we developed and estimated a logistic regression model (Table 2 and as described above). After step-wise selection, the explanatory power of the final model was weak (Tjur’s R=0.087) and school, year and gender were retained as risk factors for carriage. As expected from previous surveys of adults, the risk of carriage is higher in boys compared to girls [odds ratio (OR) 2.12, 95 % CI 1.43–3.16]. The risk of carriage was significantly lower in School 2 compared to School 1 (OR 0.5, 95 % CI 0.28–0.87) and increased for both schools in the second year of the survey (OR 2.26, 95 % CI 1.52–3.37).

**Table 2.** Risk factors associated with carriage of *S. aureus
*

Risk factor	OR	95 % CI	*P* value
**School**			
1	–	–	–
2	0.5	0.28–0.87	0.016
**Year**			
1	–	–	–
2	2.26	1.52–3.37	<0.001
**Gender**			
Female	–	–	–
Male	2.12	1.43–3.16	<0.001

### Prevalence of antimicrobial-resistance genes in colonization isolates

Isolates pooled across both schools and rounds of data collection were found to have genes associated with resistance to penicillin, erythromycin, clindamycin, fusidic acid, ciprofloxacin, meticillin, tetracycline and trimethoprim (Table 3). There was a suggestion of an increase in resistance types between rounds; however, these differences are not statistically significant within schools or with respect to the adult population represented by the isolates from the CARRIAGE study.

**Table 3.** Antimicrobial-resistance prevalence (schools estimates pooled across schools and rounds)

Drug	Schools			Community (CARRIAGE study)		
	Resistant	Total	% Prevalence (95 % CI)	Resistant	Total	% Prevalence (95 % CI)
Penicillin	296	398	75 (70–79)	263	384	68 (64–73)
Erythromycin	59	398	15 (11–19)	38	384	10 (7–13)
Clindamycin	54	398	14 (10–17)	37	384	10 (7–13)
Fusidic Acid	33	398	8 (6–11)	24	384	6 (4–9)
Ciprofloxacin	13	398	3 (2–6)	11	384	3 (1–5)
Meticillin	8	398	2 (1–4)	4	384	1 (0–3)
Tetracycline	6	398	2 (1–3)	3	384	1 (0–2)
Trimethoprim	6	398	2 (1–3)	1	384	0 (0–1)
Gentamicin	0	398	0 (0–1)	1	384	0 (0–1)

### Social network analysis

**Fig. 1.**
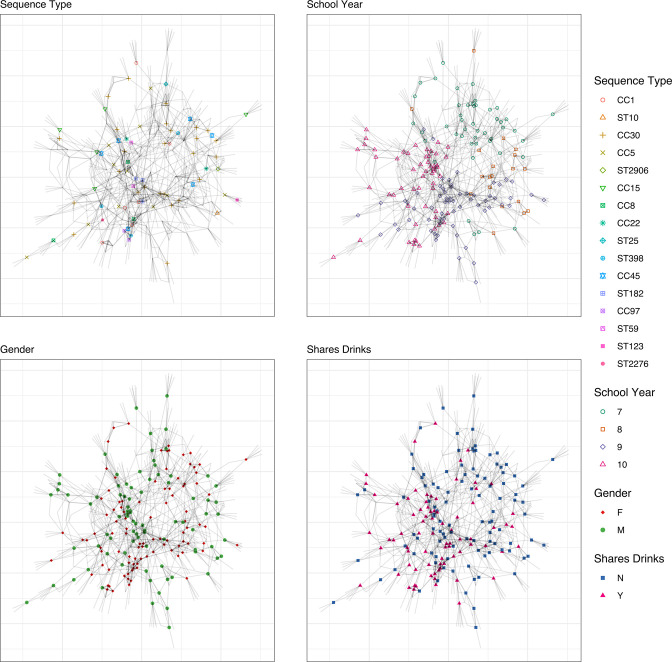
Exemplar network from School 1 (Year 1) illustrating how network structure relates to ST, school year (age of student), gender and whether students report sharing drinks with their friends. Each edge corresponds to a reported contact from a student within the study population. Nodes with no shape/colour represent named contacts within the school who did not participate in the study. Clustering with respect to school year, gender and preference to share drinks is visually evident in contrast to strain type, which demonstrates no clear pattern.

Given the unexpectedly low response rate, in particular for School 2 where effectively only a single class was surveyed in Year 2, it does not make sense to draw direct comparisons between the network structure of the two schools. For completeness, networks and measured statistics for both schools are presented in Figs S2–S6, but here we focus our discussion on School 1, illustrated by the Year 1 networks (Fig. 1).

**Fig. 2.**
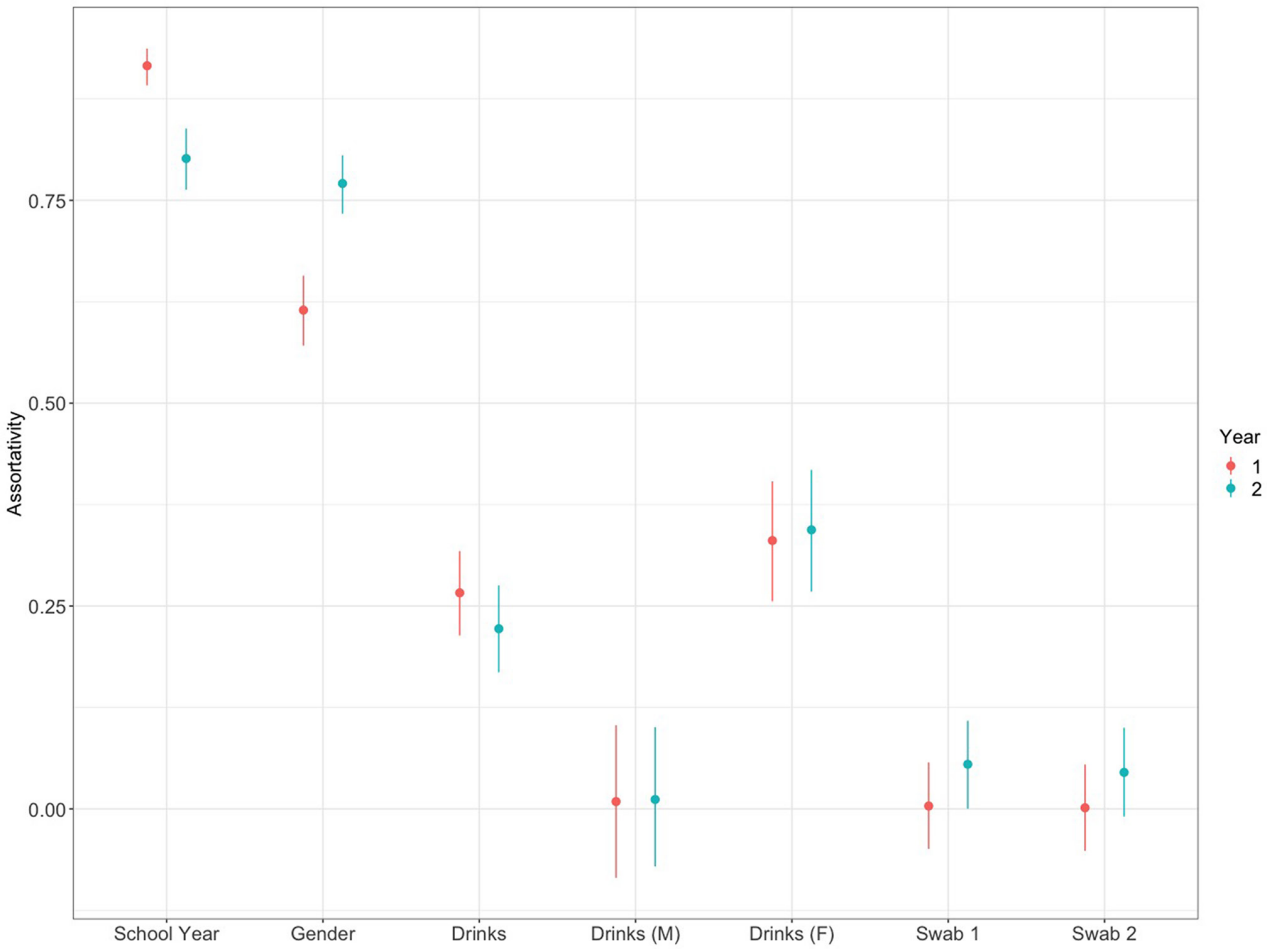
Assortativity with respect to key variables in School 1. Assortativity measures the preference of pupils to report contacts with individuals within the same school year, of the same gender, same preference for sharing drinks [which we also stratify by gender: male (M) and female (F)] and positivity for *S. aureus* (swab 1, swab 2). Lines represent 95 % CIs based on 10 000 bootstrap networks.

In our previous study of social contact networks in secondary schools, we found that self-reported contacts were stable over the course of a school year, but could vary considerably between schools [26]. Given this variation, the reported networks from School 1 are broadly consistent with our expectation, demonstrating a similar range of estimates for the clustering coefficient (0.4–0.5 95 % CI compared to 0.34–0.5; Table S1) and assortativity with respect to gender (0.57–0.81 95 % CI compared to 0.34–0.87). Contacts were also found to be much more likely to be reported within the same school year group (Figs 1 and S4), with an assortativity of between 0.76–0.94 (95 % CI; Table S2). Preference to share drinks with friends was also found to be associated with reported contacts (Figs 1 and S5) with a (weaker) assortativity of between 0.22–0.32 (95 % CI). However, considering the network of boys only, this relationship disappears with an assortativity that is indistinguishable from zero (Fig. 2, Table S2). The effect of drink sharing is entirely driven by girls, for whom the assortativity is estimated to be between 0.26–0.42 when considered alone.

In contrast to these social factors, we found no relationship between colonization status and the network structure with an assortativity for both swabs statistically indistinguishable from zero (95 % significance level). Given the duration of carriage for *S. aureus *, in itself this was not surprising and indeed was the motivation for performing transmission analysis based on whole-genome sequences of the isolates.

### Genomic epidemiology

**Fig. 3.**
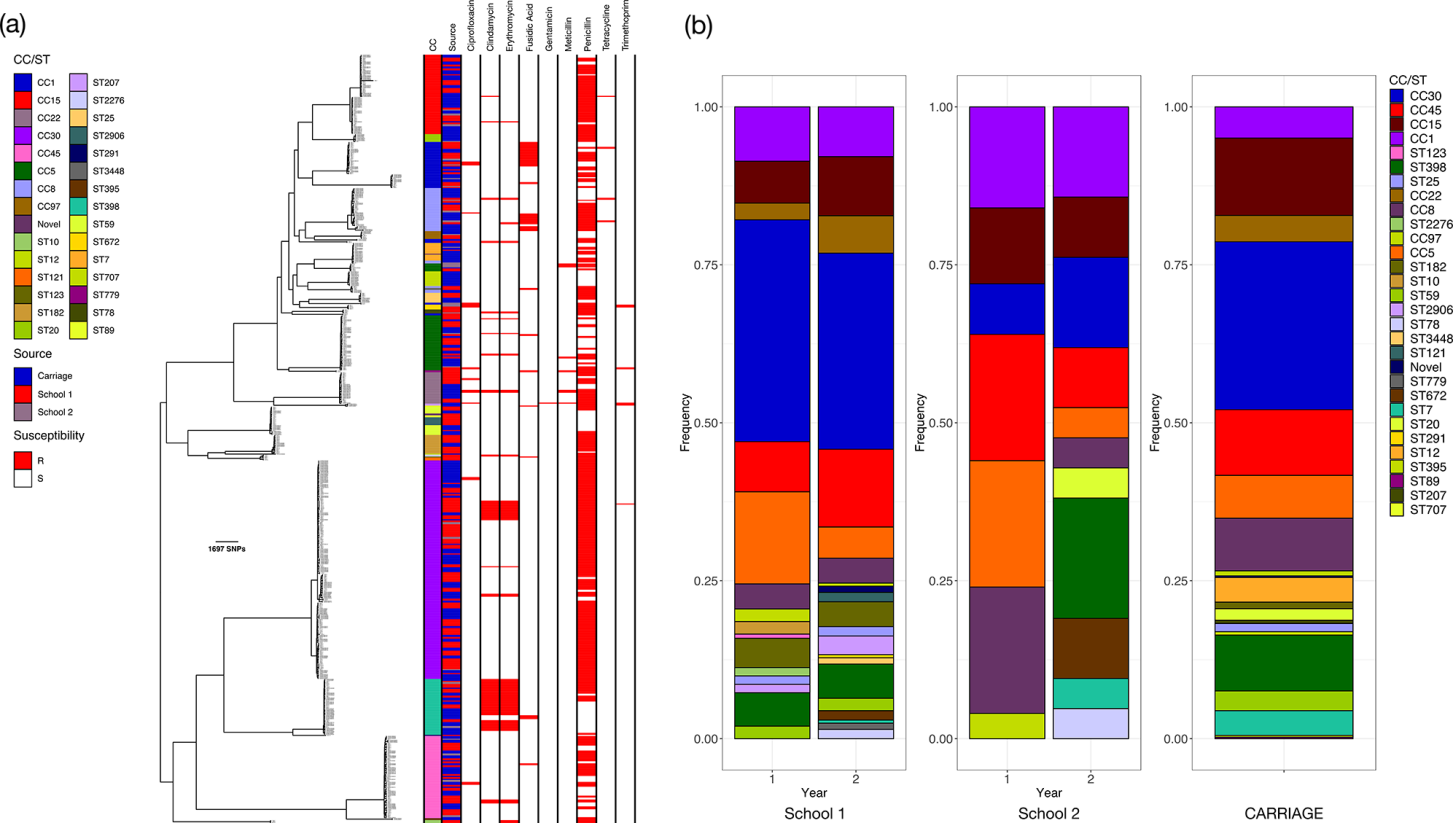
Population structure of *S. aureus* isolates. (**a**) Maximum-likelihood phylogenetic tree of 400 *S. aureus* isolates from schools and a supplemental data set of 384 isolates from the CARRIAGE study. *In silico* predicted antimicrobial-resistance determinants are shown for 9 antimicrobials; isolates positive for the determinant are highlighted in red. (**b**) Distribution of STs per year in School 1 and School 2 and distribution of STs in the CARRIAGE isolates.

A total of 400 isolates from 241 students from both schools were successfully sequenced and passed quality control. Examination of the phylogenetic tree showed that there was no school-specific clustering, with the smaller number of isolates from School 2 distributed throughout the phylogeny (Fig. 3a). Examination of the distribution of STs for each school showed that the composition of lineages circulating in each school was different (Fig. 3). In the School 1 cohort, a total of 24 unique STs were identified in Year 1 with little difference observed between the first and second swabs, whilst in Year 2, 31 unique STs were seen across both swabs (Fig. 3b). Due to the smaller dataset, fewer STs (*n*=18) were seen in the School 2 dataset, with the distribution of STs changing between the 2 years and between the two swabs in each year (Fig. 3b). Once again there was no systematic relationship found between network structure and ST (Figs 1 and S6).

### Integrated social and genomic transmission network

**Fig. 4.**
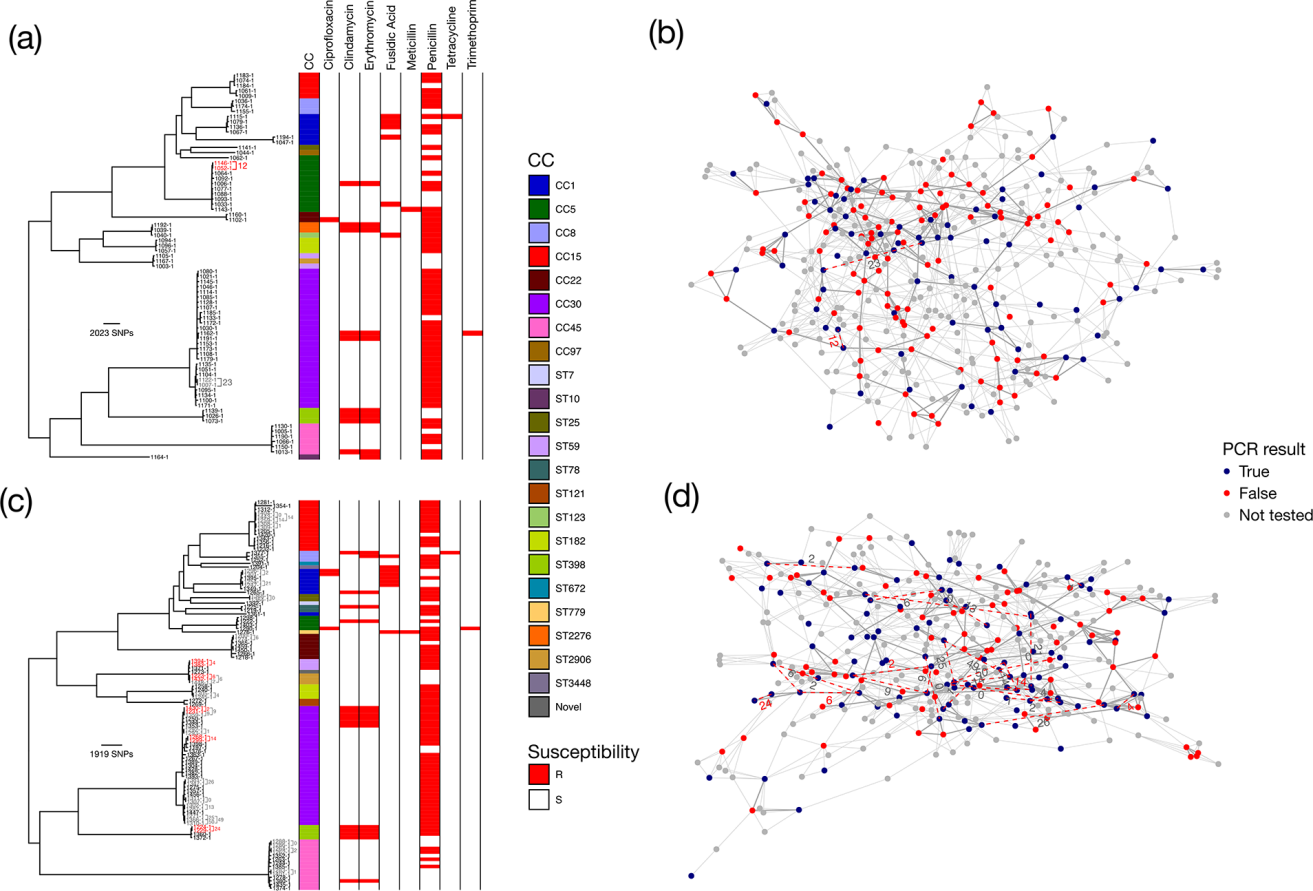
Integrated social and genomic networks for School 1. (**a**) Maximum-likelihood phylogenetic tree of 75 isolates collected from the first swab in Year 1. (b) Integrated social and genomic network for Year 1. (c) Maximum-likelihood phylogenetic tree of 108 isolates collected from the first swab in Year 2. (**d**) Integrated social and genomic network for Year 2. Putative genomic links are coloured in grey; genomic links confirmed by social links are coloured red.

Due to the small number of available sequenced isolates from School 2, genome-based transmission analysis was only examined using the isolates collected from the first swab and social networks from Year 1 and Year 2 at School 1. Putative transmission clusters calculated using a pairwise SNP threshold of 50 SNPs were overlaid on top of the social networks to attempt to identify potential routes of transmission that were supported by genomic information (Fig. 4). In Year 1, two putative genomic transmission events were identified with only one of these supported by a connection in the social network (Fig. 4a, b), whilst in Year 2, 28 putative transmission events were identified (Fig. 4c, d). Of the 28 transmission events in Year 2, 5 were linked by connections in the social network (Fig. 4d). Only 3 of the 28 transmission clusters in Year 2 contained isolates from more than two students, with each of these containing isolates from three students (Fig. 4c).

### School and CARRIAGE isolates

Due to the small number of observed direct transmissions between isolates collected at School 1, an additional 384 isolates collected in or around Cambridge as part of the CARRIAGE study were sequenced to investigate whether community transmission was contributing to *S. aureus* carriage in students at both schools. A new phylogenetic tree containing 784 isolates from both schools and the CARRIAGE study was reconstructed; this showed that all of the major lineages contained isolates from both sources (Fig. 3a). The pairwise SNP distances between isolates from either school and adults in the local area were calculated; the distributions of SNP distances were nearly identical regardless of which isolates were being compared (Fig. S7). The minimum pairwise SNP distance between any School 2 and adult colonization isolates was 55 SNPs (median 17 961 SNPs), whilst the corresponding distance for School 1 and CARRIAGE isolates was 54 SNPs (median 17 563 SNPs). Applying the same SNP threshold of 50 SNPs used earlier, no putative transmission events between students and members of the wider community were identified. Broadly similar distributions of STs were observed in the schools as in the community, with the same predominant lineages (CC1, CC15, CC30, CC45; Fig. 3b) being found.

## Discussion

In this work, we carried out a carriage study of *S. aureus* in two English secondary schools with a view to addressing two key objectives: firstly, to quantify the level and risk factors for *S. aureus* colonization in this understudied population, and secondly, to attempt to use transmission networks inferred from genomic data to validate the use of self-reported social contact data as a proxy measure for the risk of transmission.

To optimize participation rates within schools, this project was designed and carried out using a citizen science model that had previously delivered exceptionally high recruitment rates upwards of 90 % [23, 25, 26] for a broad range of secondary and primary schools distributed widely across England and Wales. In contrast, for this project, we only managed to achieve recruitment rates of between 17 and 37 % from our two schools, despite a higher level of researcher engagement and number of in-site visits (previous projects were run almost entirely remotely by video-conference). Changes to the experimental protocol to allow for online consent and offering incentives to students in the form of a prize draw improved response in one school (by 14%) but resulted in lower recruitment rates in the second (by 17%).

This experience highlights that a single approach may not be optimal for all schools, but also reflects the differences in capacity to support the project between the two schools. In School 1, we were able to pay to provide supply cover to facilitate activities within the school outside of in-contact time with the research team. Teachers’ time to be involved and support extra-curricular activities is increasingly at a premium – a key lesson learnt from this study is the necessity to budget for such additional support for teachers to provide supply cover to facilitate activities within the school outside of in-contact time with the research team. However, our recruitment rates do look more favourable when put into context of the recent COVID-19 School Infection Survey, which used similar methods and only managed to achieve participation rates of between 14 and 17 % in secondary schools across six rounds of testing [51]. It is, therefore, possible that there is simply a baseline natural level of willingness to participate in this form of study no matter the incentives, which should be considered when planning similar studies in the future.

The preference of pupils to share drinks with their friends was included in the study questionnaire on the suggestion of the citizen science teams within the schools as a proxy for the ‘riskiness of transmission’. We found no association between this hygiene-related preference and the carriage of *S. aureus *, but we did find an association with the structure of the reported networks; girls were more likely (assortativity for School 1, Year 1 of 0.33, 0.26–0.40 95 % CI) to report contacts with other girls who share the same preference. While not directly of relevance for the prediction of transmission risk, this observation suggests that hygiene preferences may be a factor in the formation of social ties within schools. However, the association could equally well arise through the reinforcement or modulation of an individual's hygiene preferences within social groups.

Prevalence of carriage of *S. aureus* in School 1 was found to be significantly higher (57–71 %, 95 % CI) than previously reported estimates from adults of ~32 % [13, 14] and with respect to estimates from the sympatric adult population of 36 % (35–37 %, 95 % CI). Seasonality is unlikely to have been a factor, as sampling was carried out at a similar time for both schools in both years. While the increased carriage prevalence with respect to adults was expected, the difference between the two schools and the increase in carriage prevalence between years is more difficult to explain. This difference was all the more surprising given that although the two schools were picked for the geographical separation, it became clear when working with them that the catchment areas of the two schools were in fact very similar. To assess whether the distribution of strain types within each school differed, we compared the distribution of observed counts of each strain in each round of sampling against the expected frequencies from the general population (i.e. the CARRIAGE data set) using a multinomial test. The multinomial test sets out to test the null hypothesis that a set of categorical count data is consistent with a set of assumed theoretical frequencies – in this case the strain frequencies from the CARRIAGE data. For each school sample set, we found no evidence of a statistically significant difference from the expectation of the CARRIAGE data (all *P* values>0.6).

The low frequency of putative transmission clusters within schools precluded a formal assessment of links between genomic and self-reported social networks. Together with the lack of evidence of clustering of lineages within schools and homogenous distribution of isolates from the school populations within the combined tree with isolates from the wider adult community (Fig. 3), we must conclude from our data that transmission of *S. aureus* within schools is simply too rare to make it a viable instrument for tracking modes and rates of transmission within a school community.

To explore the representativeness of our school isolates for the general community, we sequenced an additional data set of isolates from the CARRIAGE study carried out in adults living in the same Cambridgeshire local community. Isolates from both the school and adult populations clustered together on the combined phylogenetic tree, suggesting that our school populations are representative of the diversity of *S. aureus* found in the general community, with indistinguishable levels of antimicrobial resistance (Table 3).

School-age children are an understudied group with respect to the colonization and transmission of *S. aureus *. Despite the issues with recruitment, to our knowledge this study still constitutes the largest systematic study to date on the prevalence of colonization and diversity of *S. aureus* in school-age children in the UK [15]. While we found no evidence that schools themselves are important as a route of transmission, the increased rates of colonization, compared to the local community, found within schools indicate that school-age children may be an important source of transmission within the community in other contexts.

## Supplementary Data

Supplementary material 1

Supplementary material 2
