# Supplementary material for: Colonization and transmission of Staphylococcus aureus in schools: a citizen science project
Source: Microb Genom. 2023 Apr 19;9(4):mgen000993. doi: 10.1099/mgen.0.000993 (PMC10210949; doi:10.1099/mgen.0.000993)
Supplement: Supplementary material 1 [file mgen-9-993-s001.pdf]

Table S1: Comparison of network statistics between schools (95% confidence intervals based on 10,000 bootstrap simulations)

Characteristic	School 1, Year 1<br>Median (95% CI)	School 1, Year 2<br>Median (95% CI)	School 2, Year 1<br>Median (95% CI)	School 2, Year 2<br>Median (95% CI)
Clustering Coefficient	0.412 (0.394, 0.429)	0.425 (0.401, 0.445)	0.494 (0.453, 0.509)	0.4091 (0.4091, 0.4091)
Reciprocity	0.468 (0.435, 0.502)	0.445 (0.410, 0.478)	0.43 (0.37, 0.50)	0.55 (0.45, 0.64)
Mean Distance	6.93 (6.10, 7.65)	8.05 (7.04, 9.31)	3.23 (2.80, 3.45)	2.0200 (2.0200, 2.0200)
No. of Communities	37.0 (30.0, 43.0)	29.00 (24.00, 35.00)	13.00 (11.00, 15.00)	12.00 (9.00, 13.00)
Community Size	61.4 (51.5, 74.1)	61.1 (53.8, 69.9)	18.38 (16.73, 21.07)	7.50 (7.50, 8.60)

Table S2: Assortativity of social networks measured in School 1 with respect to key characteristics (95% confidence intervals based on 10,000 bootstrap intervals)

**Characteristic**	Year 1 (95% CI)	Year 2 (95% CI)
Assortativity (Year)	0.92 (0.89, 0.94)	0.80 (0.76, 0.84)
Assortativity (Gender)	0.61 (0.57, 0.66)	0.77 (0.73, 0.81)
Assortativity (Shares Drinks)	0.27 (0.22, 0.32)	0.22 (0.17, 0.28)
Assortativity (Shares Drinks M)	0.01 (-0.08, 0.11)	0.01 (-0.07, 0.10)
Assortativity (Shares Drinks F)	0.33 (0.26, 0.40)	0.34 (0.27, 0.42)
Assortativity (Swab 1)	0.00 (-0.05, 0.06)	0.05 (0.00, 0.11)
Assortativity (Swab 2)	0.00 (-0.05, 0.06)	0.04 (-0.01, 0.10)

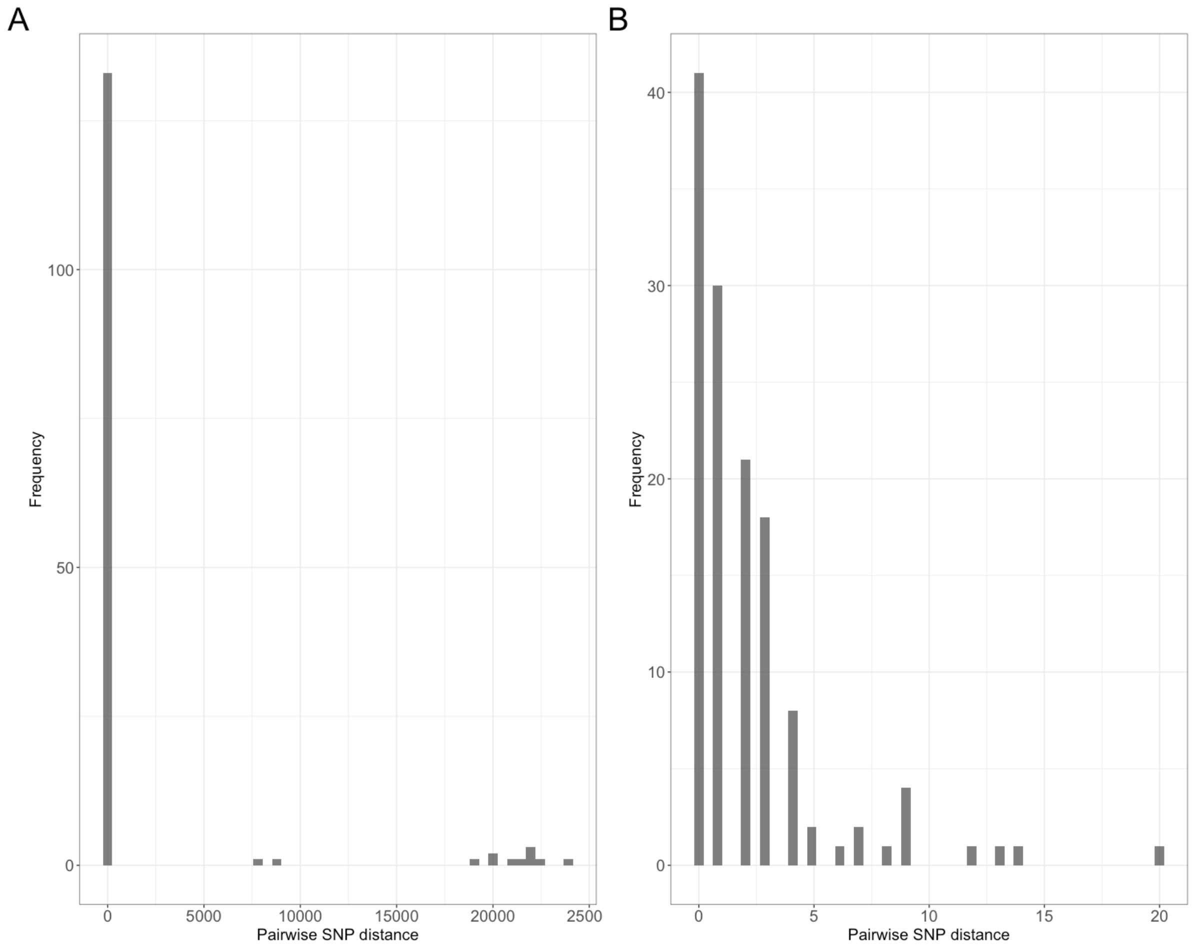

Figure S1: Within-student diversity of isolates collected from students at School 1. A) Histogram of per student pairwise SNP distances calculated using isolates collected at both sampling points in Year 1. B) Zoomed in plot of panel B only showing pairwise SNP distances less than 25 SNPs.

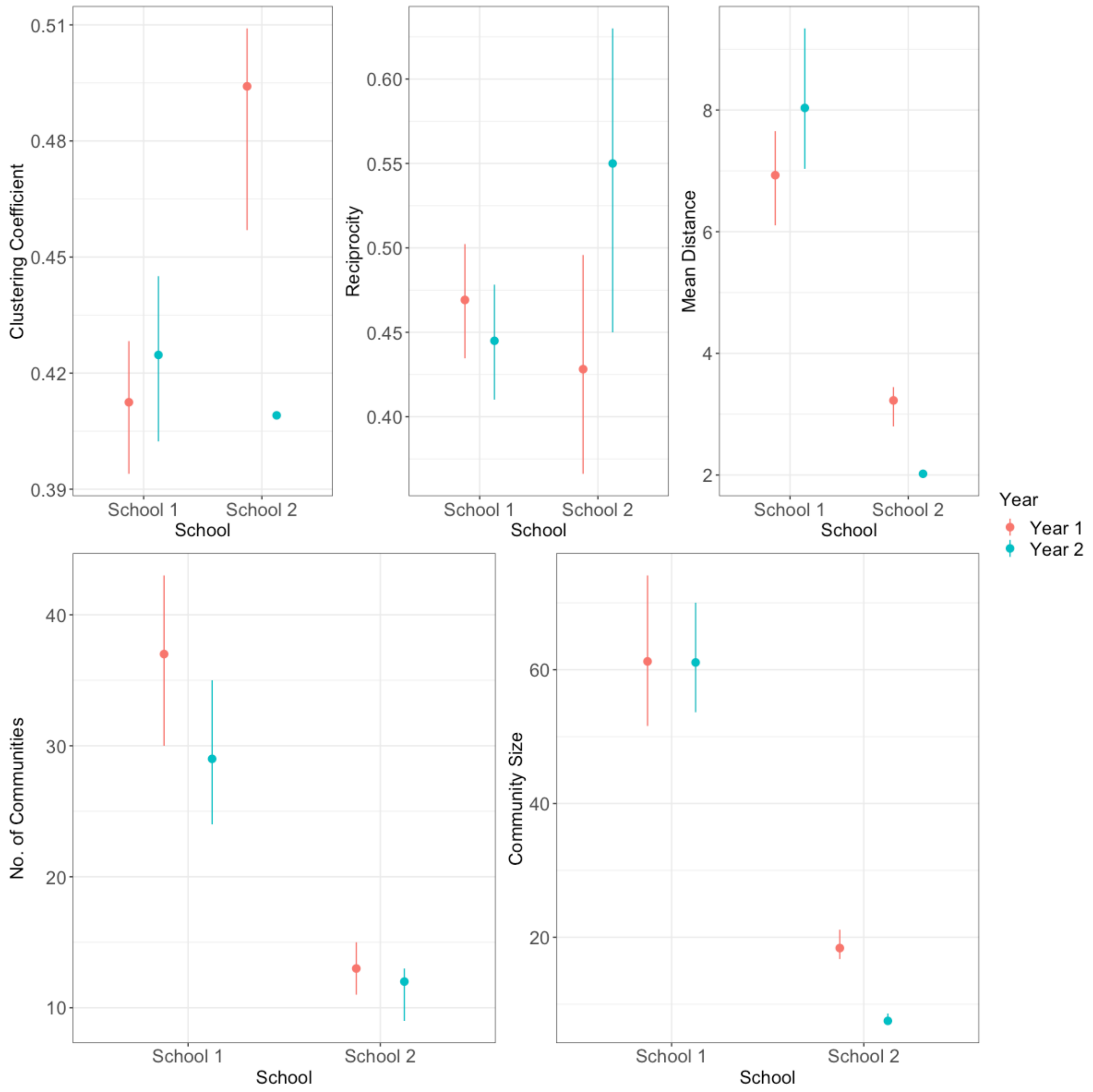

Figure S2: Comparison of summary network statistics between schools and year of study. As described in the main manuscript we characterise the structure of measured social networks within each school and year of survey in terms of the clustering coefficient (transitivity), reciprocity (fraction of pupils that mutually name each other), mean distance between pupils, number of communities and size of communities as calculated by the “walktrap” algorithm. Uncertainty is represented by 95% bootstrap confidence intervals (lines). Broadly, network characteristics were consistent between the two years of study with the exception of the clustering

coefficient measured for School 2. However, interpretation of this difference or any of the measured differences between schools is precluded by the exceptionally low recruitment rate in School 2 - essentially limited to a single class in the second year.

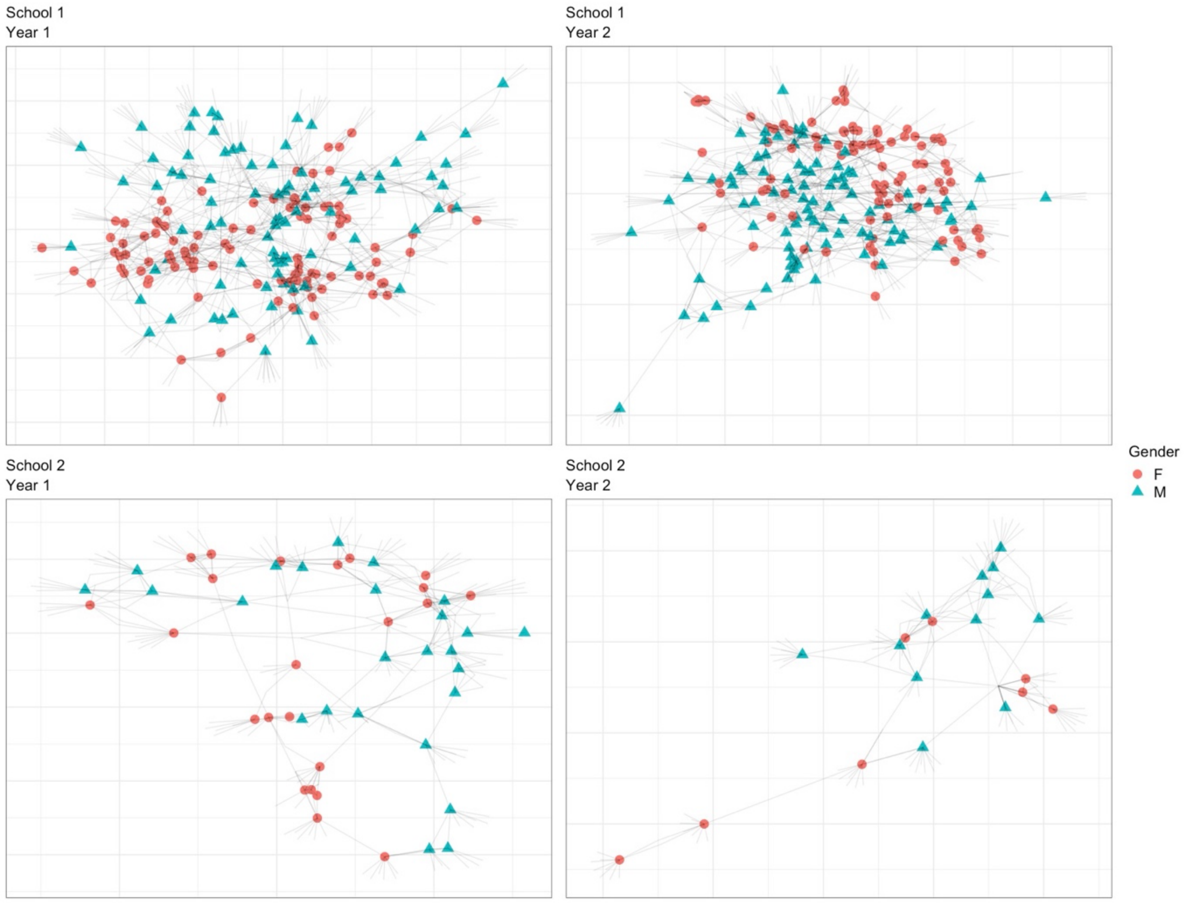

Figure S3: All measured social networks demonstrate clear segregation by gender.

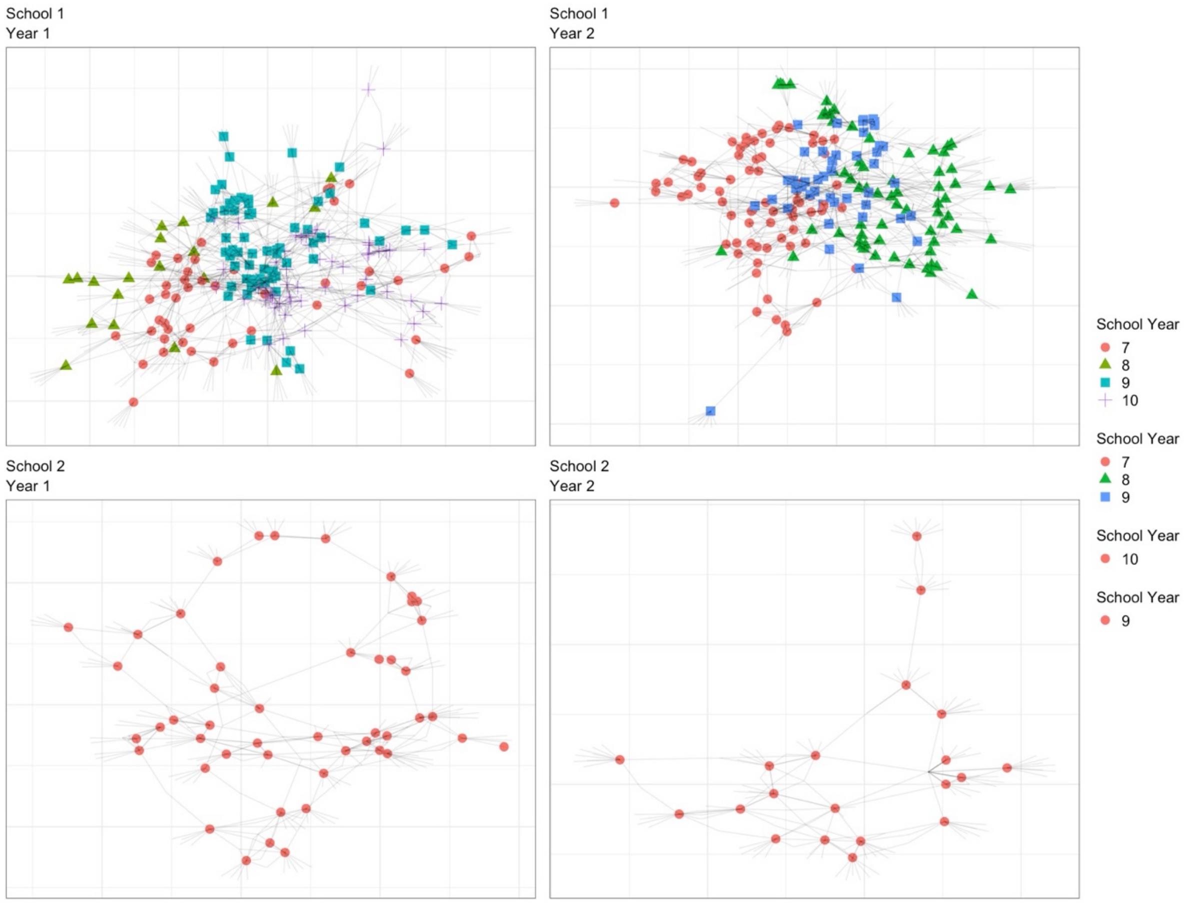

Figure S4: All measured social networks demonstrate strong patterns of segregation by school year group.

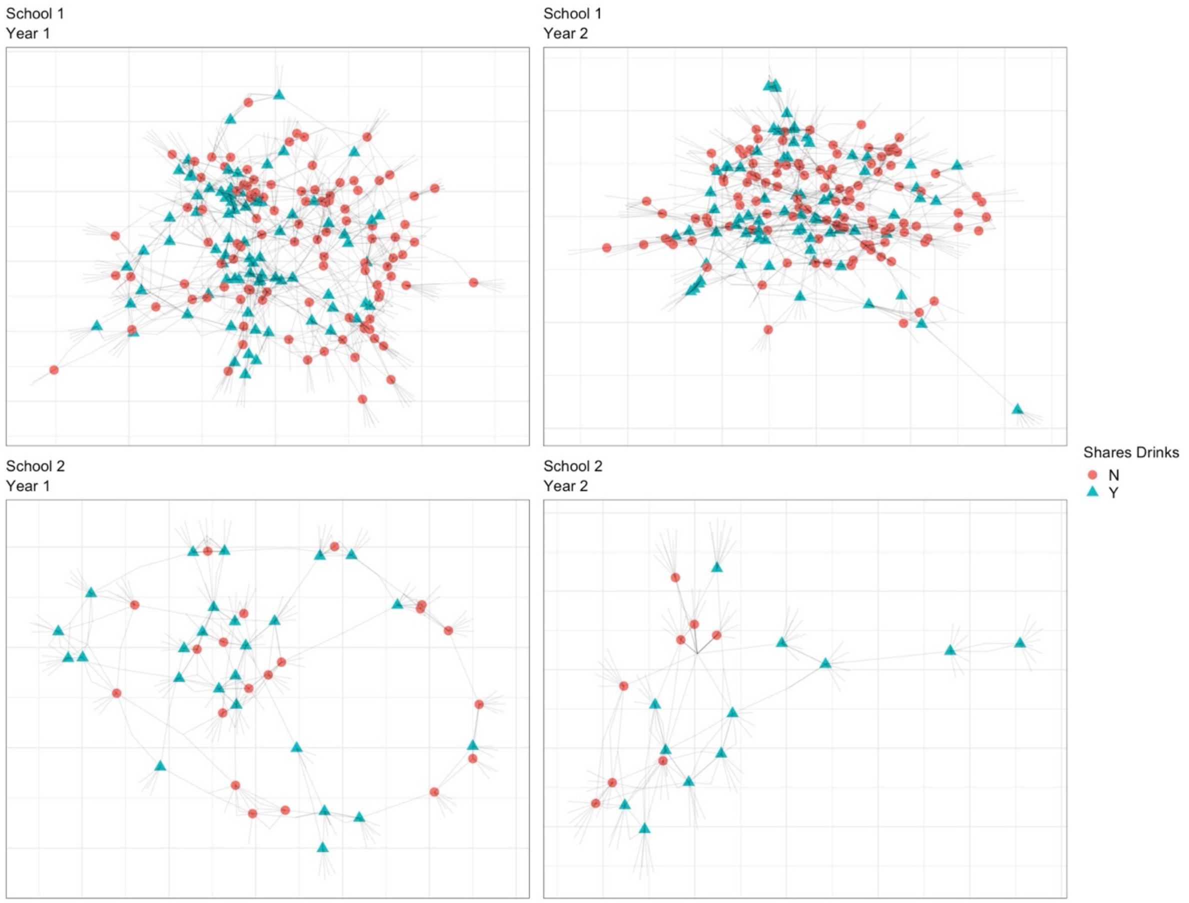

Figure S5: All measured social networks suggest segregation by preference to share drinks with friends.

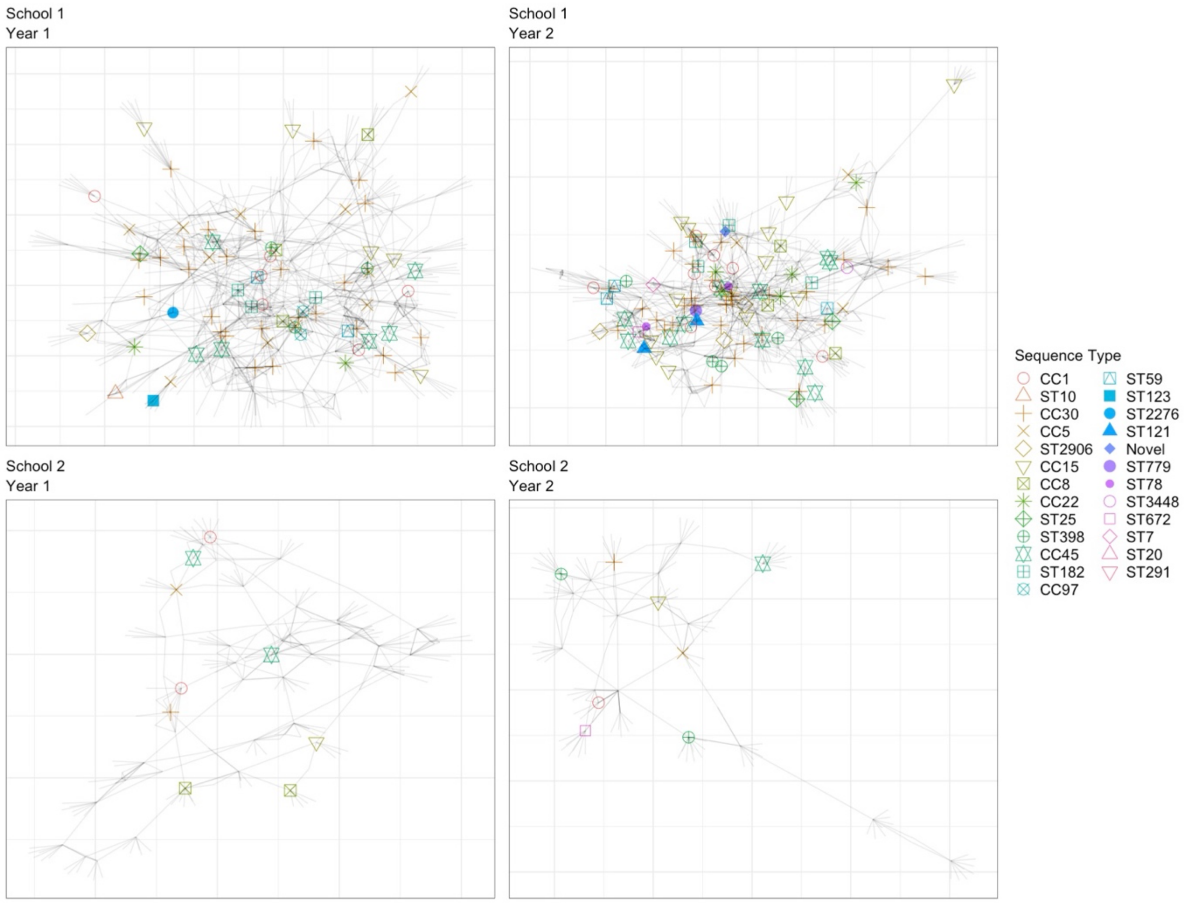

Figure S6: Distribution of strain types on measured social networks by school and year of study. Strain type demonstrates no clear association with self-reported social networks.

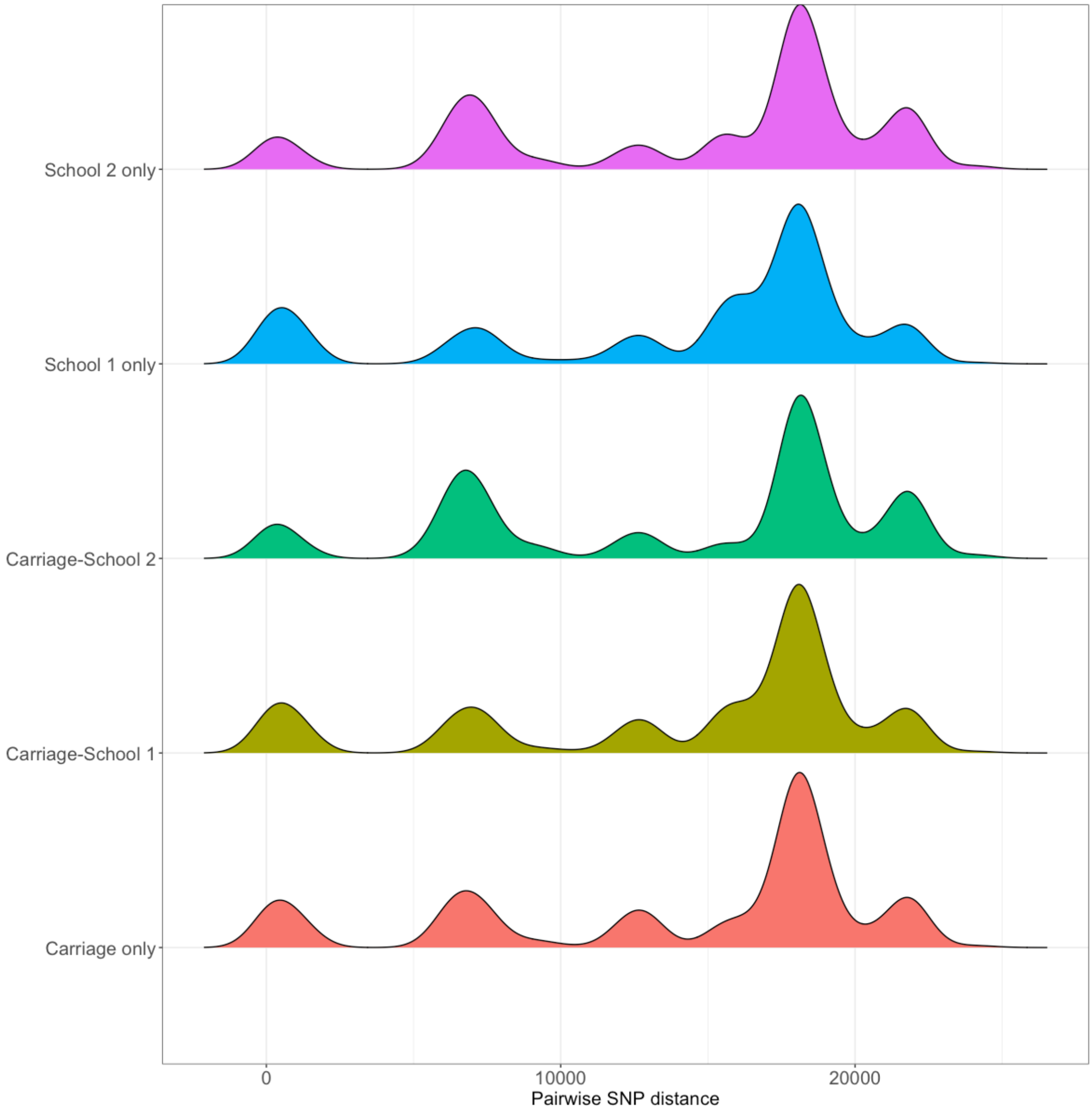

Figure S7: Comparison of pairwise SNP distances between school and carriage isolates
